# An unbiased stereological method for corneal confocal microscopy in patients with diabetic polyneuropathy

**DOI:** 10.1038/s41598-020-69314-2

**Published:** 2020-07-28

**Authors:** Ellen L. Schaldemose, Rasmus E. Hammer, Maryam Ferdousi, Rayaz A. Malik, Jens R. Nyengaard, Páll Karlsson

**Affiliations:** 1 Danish Pain Research Center, Aarhus University Hospital, Aarhus, Denmark; 2 Department of Clinical Medicine – Core Centre for Molecular Morphology, Section for Stereology and Microscopy, Aarhus University, Aarhus, Denmark; 3 Centre for Endocrinology and Diabetes, Institute of Human Development, Manchester Academic Health Science Centre, Manchester, UK; 4 Division of Medicine, Weill Cornell Medical College in Qatar, Doha, Qatar; 5 Centre for Stochastic Geometry and Advanced Bioimaging, Aarhus University Hospital, Aarhus, Denmark

**Keywords:** Neuropathic pain, Diabetes complications

## Abstract

Corneal confocal microscopy (CCM) derived corneal nerve measures are lower in diabetic sensorimotor polyneuropathy (DSPN). There are, however, methodological challenges in relation to adequate and unbiased sampling of images with objective corneal nerve quantification. Here we compare a new sampling method and adjusted area calculation with established methods of corneal nerve quantification in patients with and without DSPN and healthy controls. CCM images from 26 control subjects and 62 patients with type 1 diabetes with (n = 17) and without (n = 45) DSPN were analyzed. The images were randomly selected and corneal nerve fiber length (CNFL), corneal nerve fiber branch density (CNBD) and corneal nerve fiber density (CNFD) were determined in both a manual and automated manner. The new method generated 8–40% larger corneal nerve parameters compared to the standard procedure (*p* < 0.05). CNFL was significantly reduced using the new method for both manual and automated analysis; whilst CNFD and CNBD were significantly reduced using the automated method in both diabetic groups compared with controls. The new, objective method showed a reduction in corneal nerve parameters in diabetic patients with and without DSPN. We recommend using a randomized sampling method and area-dependent analysis to enable objective unbiased corneal nerve quantification.

## Introduction

Corneal confocal microscopy (CCM) is a non-invasive ophthalmic technique that has been used to quantify corneal nerve fibers as a surrogate for small fiber pathology (SFP) in a number of peripheral neuropathies including diabetic sensorimotor polyneuropathy (DSPN)^1–6^. Compared to skin biopsy, the “pathological gold standard” for diagnosing SFP, CCM has some advantages, including rapid, non-invasive nerve imaging and analysis^7–9^. Both manual and automated analysis, and more recently artificial intelligence-based algorithms have been used to show good sensitivity and specificity for the diagnosis of DSPN^1,10–13^. However, there are methodological challenges in relation to adequate sampling of images across the cornea and objective nerve quantification. From approximately 100 images collected during a bilateral CCM procedure three to eight images are subjectively selected using criteria based on image quality and the presence of in-focus nerve fibers, which may introduce selection bias influencing the final assessment^11,14,15^. To address this limitation we have developed a new randomized sampling method^16^. Additionally, it is still difficult to capture in-focus nerve fibers across the whole image^17^. To ensure the use of only the focused images, we have developed a method to delineate the specific area of the images where the nerves are in focus. Hereby it is possible to calculate the nerve characteristics within an adjusted in-focus area^16^. In the aforementioned methodological study, we demonstrated that the corneal nerve fiber length was numerically higher than the conventional method in patients with idiopathic small fiber neuropathy^16^. However, it is unclear whether this increase differs between healthy controls and patients. In this study, we have compared the new sampling method and adjusted area calculation with established corneal nerve and skin biopsy quantification in well-characterized patients with and without DSPN and healthy controls.

## Results

A summary of the demographic data of the participants obtained by Chen et al. is presented here^1^: The mean age was significantly higher in the DSPN( +) group compared to both the control and the DSPN(-) group, *p* < 0.001 (59 ± 11 years vs 44 ± 15 years and 44 ± 13 years, respectively) and the duration of diabetes was significantly longer in the DSPN( +) group (39 ± 14 years vs 23 ± 15 years). Neuropathy disability score was significantly higher in the DSPN( +) group compared to controls. Vibration perception threshold was significantly higher in the DSPN( +) group compared to both DSPN(-) and control (25.2 ± 13.4 V (DSPN( +)) vs. 7.6 ± 5.5 V (DSPN(-)) and 6.0 ± 5.5 V (control)), *p* < 0.001). Similarly, the peroneal motor nerve conduction velocity was lower in both diabetic groups compared to control and lowest in the DSPN( +) group (31.0 ± 9.5 m/s (DSPN( +)) vs 43.9 ± 3.1 m/s (DSPN(-)) vs 49.1 ± 3.4 m/s (control), *p* < 0.001).

### CCM parameters with the new method

#### Manual CCM analysis

Using the randomized and area adjusted method, CNFD and CNFL were significantly reduced in the DSPN( +) group compared to both the control and the DSPN(-) group. In contrast, the CNFL values were larger in the DSPN(-) group compared to the healthy controls. There were no differences in CNBD between the groups (see Supplementary Table S1 and Supplementary Fig. S1, A online).

#### Automated CCM analysis

For all CCM parameters (CNFD, CNFL and, CNBD) the values were significantly reduced in the DSPN( +) and DSPN(-) group compared to the controls and were lowest in the DSPN( +) group (see Supplementary Table S2and Supplementary Fig. S1, B online).

#### Unadjusted versus adjusted area, randomized sampling method

 The adjusted area increased the actual CCM values by 35–64%. There was a strong correlation between the unadjusted and the adjusted area CCM parameters with Pearson’s correlation coefficients between r = 0.87 to r = 0.93 (see Supplementary Table 2 and Supplementary Fig. S2, A and B online). The relative increase in CNFL (both automated and manual analysis) when using the adjusted area was significantly lower in the control group (Kruskal Wallis test, *p* < 0.05, unpaired t-test, *p* < 0.05) compared to both the DSPN(-) and the DSPN( +) group. Likewise, for the CNFD and CNBD measurements, when using the adjusted area, there were tendencies for a higher relative increase in the diabetic groups compared to the control group (Kruskal Wallis test, *p* values from *p* = 0.06 to *p* = 0.4). There were no differences between the diabetic groups (CNFL: *p* = 0.052, manual, *p* = 0.08 automatic, unpaired t-test, CNFD and CNDB *p* > 0.05, Kruskal Wallis test).

### New method compared to skin biopsy

**Figure 1:**
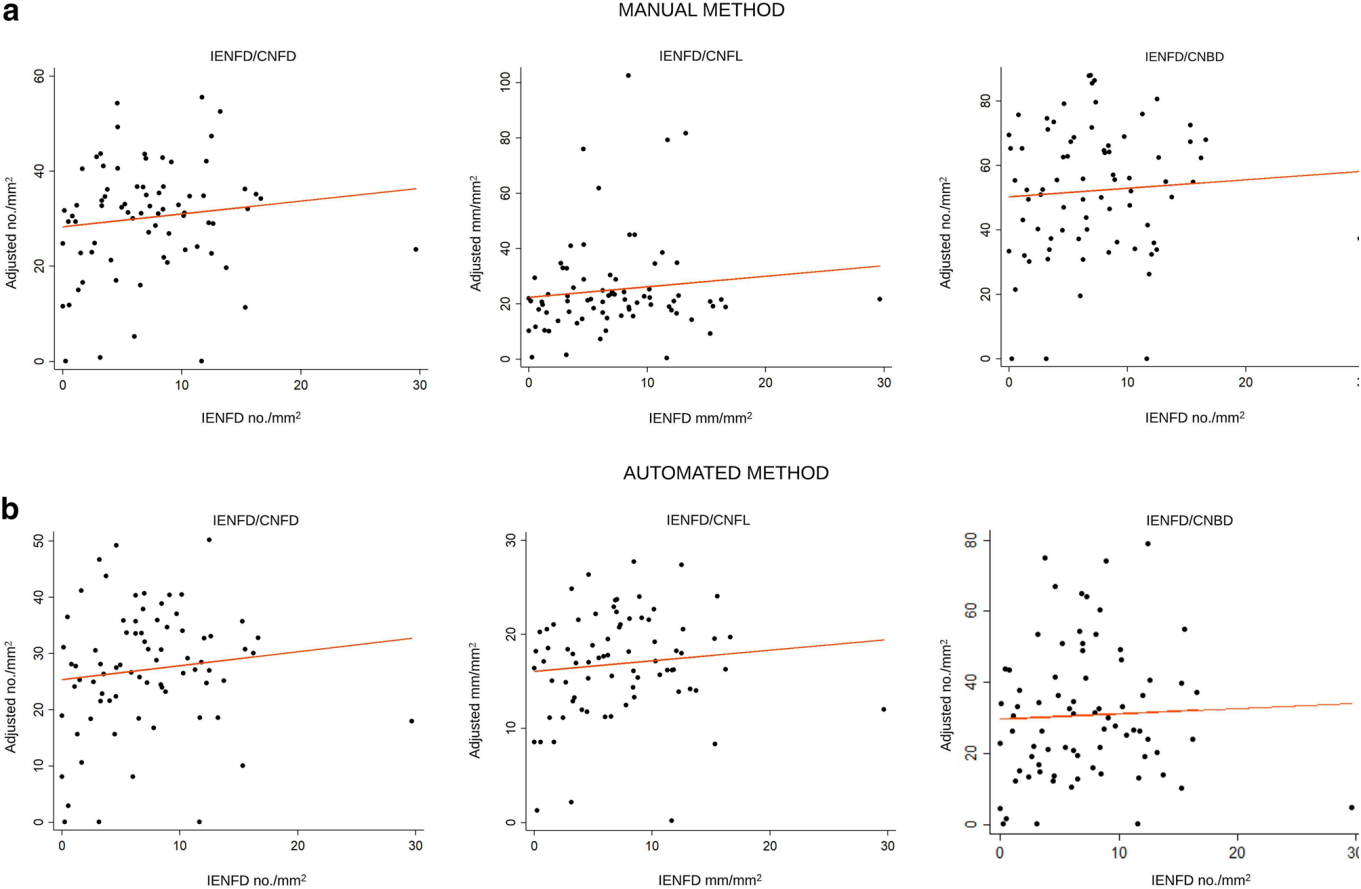
Scatter plot and regression line of IENFD vs CCM results using the area adjusted and randomized sampling method, grouped by method of analysis (**A**: manual, **B**: automated). The dots represent the mean values from the individual participants and the dashed lines indicate the regression line. The Pearson’s correlation coefficients were r = 0.12 (manual method) and r = 0.13 (automated method) for the CNFD values, r = 0.11 (manual method) and r = 0.10 (automatic method) for the CNFL values and r = 0.066 (manual method) and r = 0.038 (automated method) for the CNBD values.

**Figure 2:**
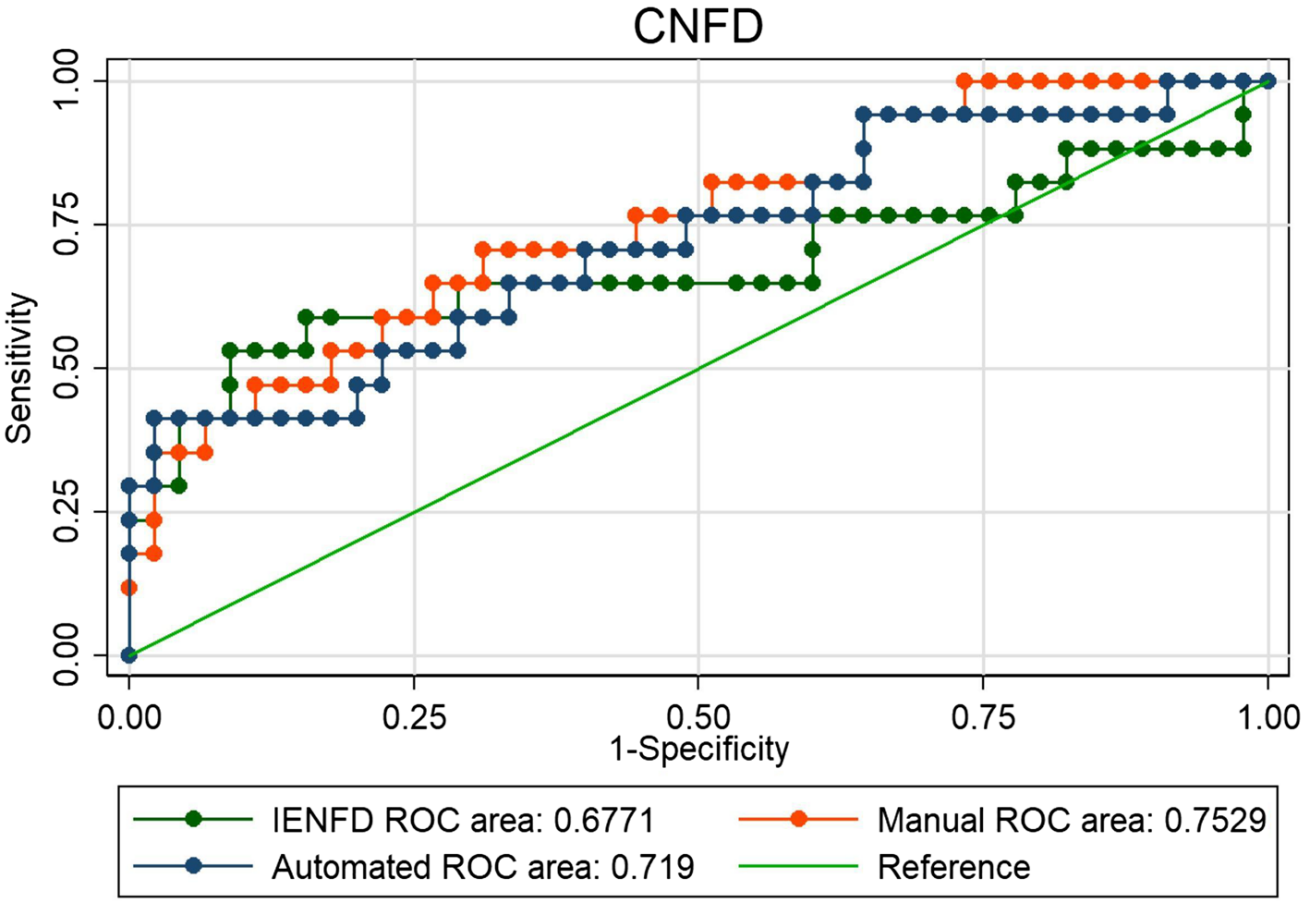
ROC curve. AUC between 0.68 and 0.75, no statistical difference between the three curves, chi^2^ = 1.16, *p* = 0.56.

There was no correlation between the randomized sampling method and area adjusted method and IENFD, Pearson’s correlation coefficients between r = 0.04 and r = 0.13, respectively (Fig. 1). Likewise, there was no correlation between IENFD and the CCM values originally reported by Chen et al. (manual r = 0.17 (CNFL), r = 0.13 (CNBD), r = 0.23 (CNFD) and automated: r = 0.17 (CNFL), r = 0.10 (CNBD), r = 0.14 (CNFD)). Figure 2 presents a representative example of the ROC curves (CNFD and IENFD values). The AUC’s for DSPN were between 0.63 and 0.75 with no statistically significant differences between the two methods (CNFD; chi^2^ = 1.16, *p* = 0.56, CNFL: chi^2^ = 5.64, *p* = 0.06 and CNBD: chi^2^ = 3.16, *p* = 0.21).

### New method compared to the standard method

To secure an equal analysis method, only the automated analyses were compared. The randomized sampling and adjusted area method generated numerically (8–40%) larger CCM parameters (Table 1) compared to the standard procedure (*p* < 0.05, paired t-test), except for CNFD in the control group.

**Table 1:** Randomized sampling and adjusted/unadjusted area versus standard method with automated analysis.

	New, randomized sampling		Standard^1^	% mean difference	
	Adjusted area (mean ± SD)	Unadjusted area (mean ± SD)	(Mean ± SD)	Adjusted area vs standard method	Unadjusted area vs standard method
**Control (n = 26)**					
CNFD no./mm^2^	33.7 ± 5.6	25.1 ± 4.88	31.3 ± 6.5	7.7%	− 0.2%*
CNFL mm/mm^2^	20.5 ± 3.5	15.2 ± 2.87	17.7 ± 2.8	15.8%*	− 0.1%*
CNBD no./mm^2^	44.3 ± 18	33.3 ± 14.2	44.6 ± 17	** − **0.7%	− 0.3%*
**DSPN( −) (n = 45)**					
CNFD no./mm^2^	28.2 ± 9.3	20.4 ± 1.13	22.6 ± 7.3	24.8%*	− 0.1%*
CNFL mm/mm^2^	17.0 ± 4.2	12.2 ± 0.524	13.4 ± 3.3	26.9%*	− 0.1%*
CNBD no./mm^2^	31.1 ± 18	22.5 ± 2.06	26.2 ± 15	18.7%*	− 0.1%*
**DSPN( +) (n = 17)**					
CNFD no./mm^2^	17.3 ± 12	11.4 ± 9.11	13.5 ± 9.1	28.1%*	− 0.2%^
CNFL mm/mm^2^	12.3 ± 6.8	7.92 ± 4.64	8.8 ± 4.7	39.8%*	− 0.1%*
CNBD no./mm^2^	19.1 ± 14	12.4 ± 9.64	15.4 ± 12	24.0%^	− 0.2%^

Standard method from Chen et al.^1^ significantly larger values using the new randomized and *adjusted* area method compared to the standard method (except for CNFD and CNBD in the control group). Smaller values using the new randomized, but *unadjusted* area method compared to standard. The differences are statistically but not clinically significant.

Statistically significant differences are marked in bold, *p < 0.001; ^*p* < 0.05.

When comparing the randomized sampling method to the standard method, using the unadjusted area the CCM values were reduced compared to the standard method (*p* < 0.05, paired* t* test), but since the absolute difference was small it was not considered clinically relevant (Table 1).

### Interobserver reliability

There was no significant difference in mean between the investigator and the blinded second observer for CNFL, CNFD, and CNBD (*p* = 0.14, *p* = 0.29, *p* = 0.49, respectively, paired t-test) and correlations of variance were (investigator vs. observer) 32% vs. 45%, 39% vs. 17% and 50% vs. 48% for CNFL, CNFD, and CNBD respectively.

## Discussion

In this study, we have compared a new randomized and unbiased sampling method and area dependent analysis with standard manual and automated CCM analysis. The new method generated larger CCM parameters compared to the standard method, mostly due to adjustment of the area analyzed but showed a comparable and progressive reduction in CCM parameters in diabetic patients with and without DSPN compared to controls. The larger CNFL values in the DSPN(-) group compared to controls (manual analysis, adjusted area) is possibly due to variations in the manual analysis.

The randomized sampling method (unadjusted area) showed a slight reduction in all CCM parameters. Due to their objectivity, randomized sampling methods are recommended in general and indeed we show a progressive reduction in corneal nerve parameters in patients with and without DSPN. In this study we found no correlation between the CCM and IENFD, unlike other studies^1,10,18^. The relationship between CCM and IENFD remains somewhat unclear and needs to be delineated. Reasons for these discrepant results are unclear, but may include variations in the skin biopsy procedure (e.g. site of biopsy, fixation methods, staining protocol, section thickness and counting rules), a floor effect with IENFD with very low values in some participants and variations in study cohorts (e.g. population size, disease duration and severity). ROC analysis, however, showed that CCM using the new method and IENFD had a comparable ability to discriminate between diabetic patients with and without DSPN.

An increase in CCM parameters when using the adjusted area method is expected since the image area will be reduced due to lack of focus on the nerve layer in the whole image. There was a lower relative increase in CNFL in the control group compared to the diabetic groups, indicating that this could be a potential confounder where patient images are getting false low values. However, despite this reduction in CCM parameters the difference was maintained between patients with and without DSPN.

In conclusion, our proposed objective selection method avoids subjective selection bias when selecting CCM images but is comparable to the standard CCM image selection method in showing a reduction in corneal nerve fibers and differentiating patients with DSPN from controls. We recommend using a randomized sampling method and area dependent analysis for more objective and accurate corneal nerve quantification. The image selection process does not take additional time from current methods and the new artificial intelligence-based algorithms that have been developed will hopefully be further improved so they can also remove out-of-focus areas from the images to quickly acquire more accurate CCM measures.

## Methods

### Participants

 CCM images from 26 control subjects and 62 patients with type 1 diabetes with DSPN (n = 17, DSPN( +)) and without DSPN (n = 45, DSPN(-)) defined by the Toronto Diabetic Neuropathy Expert Group criteria^19^ from a previously published study were included^1^. DSPN was defined by the Toronto Diabetic Neuropathy Expert Group criteria^19^. The DSPN assessment including Neuropathy Disability Score (NDS), vibration perception threshold (VPT) and peroneal motor nerve conduction velocity (PMNCV) as measured in the original study^1^. The already published study and the current study were conducted according to the Declaration of Helsinki II and the original experimental protocol was approved by the North Manchester Research Ethics Committee. All methods were carried out in accordance with relevant guidelines and regulations. Informed written consent was obtained from all participants prior to enrollment.

### CCM

All CCM scans were performed by two trained investigators using The Heidelberg Retinal Tomograph with Rostock Corneal Module (Heidelberg Engineering GmbH, Germany)^14,20^. The CCM scanner generates images with a size of 380 × 380 pixels and an area of 400 × 400 µm^2^. Images from the sub-basal nerve plexus were selected. This plexus is of particular interest in neuropathy since it is the main nerve plexus of the nerves supplying the corneal epithelium^21^.

### Sampling method

**Figure 3:**
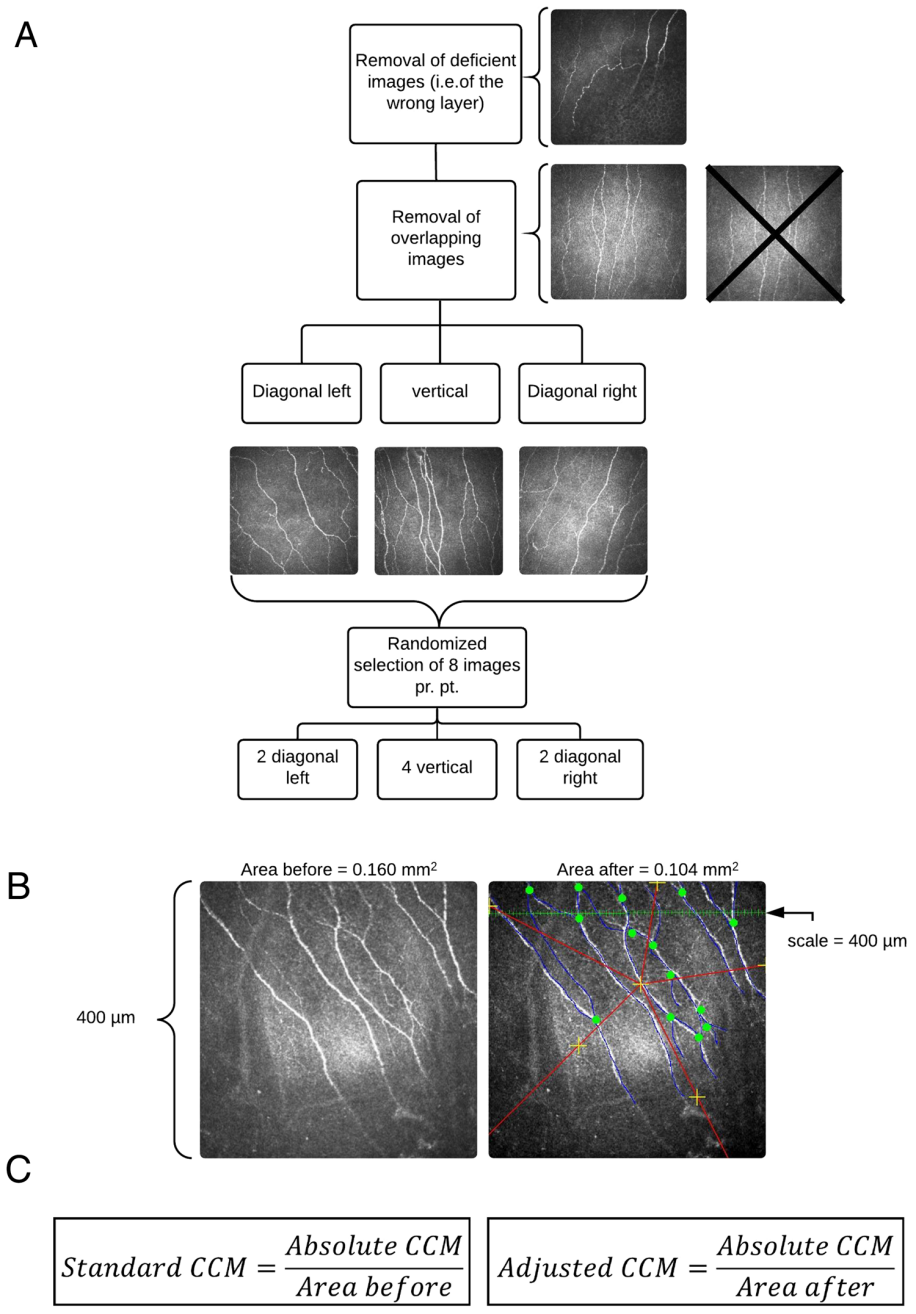
(**A**) Flowchart of the new randomized sampling method and area calculation. (**B**) Adjusted area calculation using the 2D nucleator by newCAST. Right images illustrate how to delineate the area in focus. The area_after_ is estimated as: *a*_*after*_ = *π* · *I*^2^, where *l* is the length of a test ray from the central cross to the intersection. (**C**) Calculation of the adjusted CCM value. Blue lines are identified and traced nerve fibers and green dots are identified nerve fiber branches.

 The images were randomly selected using our new method (Fig. 3A)^16^. First, to secure an equal distribution of images across the cornea, the images were divided according to the fiber orientation: vertical, diagonal left and diagonal right^21,22^. Then, eight images (four images with a vertical nerve orientation and two diagonal images with left and right orientation, respectively) were randomly selected using systematic sampling with a fixed sample size^23^. In Chen et al., five sub-basal images from the right and left eyes were selected for analysis using the criteria of depth, focus position and contrast^1^.

### Nerve fiber analysis

 The following parameters were included in the study: Corneal nerve fiber length (CNFL), defined as the total length of main fibers and branches per mm^2^, the corneal nerve fiber density (CNFD), defined as number of main fibers per mm^2^ and the corneal nerve fiber branch density (CNBD), defined as the total number of primary branches per mm^2^. The parameters were determined in both a manual and an automated manner.

### Software

 Two investigators performed the manual analysis using CCMetrics, version 1 (CCMetrics: M.A. Dabbah, Imaging Science, University of Manchester)^24,25^. The automated analyses were performed using ACCMetrics, version 2 (CCMetrics: M.A. Dabbah, Imaging Science, University of Manchester).

### Area

 The adjusted area (defined as the area of the images where the sub-basal nerve layer was in focus^16^) was estimated using the 2D nucleator (newCAST, version 6.2, (Visiopharm A/S, Hørsholm, Denmark, Gundersen, 1988). Figure 3B,C displays an overview of the calculation of the adjusted values.

### Interobserver reliability

 To determine inter-observer reliability for the quantification of CNFL, CNFD and CNBD with the manual method, eight already selected images from five participants were randomly chosen and the second and blinded investigator repeated the manual analysis.

### Statistics

 The mean of the featured CCM values for each participant was used. Stata for Windows (version 14.1) was used for data analysis. Data were visually inspected for a normal distribution using QQ-plots. To compare the three groups one-way ANOVA or Kruskal Wallis tests (for unequal data) were performed. A *p* value < 0.5 was considered statistically significant. Paired t-tests were used to calculate differences between the new and standard method. The Pearson’s correlation coefficients were determined between the related variables, i.e. between the new and standard method, the new method and results from the IENFD, and the unadjusted versus adjusted area with the randomized sampling method. The correlations were illustrated using scatter plots. Receiver operation characteristic (ROC) curves and the area under the curve (AUC) were conducted to evaluate the different methods for the diagnosis of DSPN. Chi^2^ tests were used to compare the AUC’s for each CCM parameter and IENFD.

## Supplementary information

Supplementary information.
