# Supplementary material for: An unbiased stereological method for corneal confocal microscopy in patients with diabetic polyneuropathy
Source: Sci Rep. 2020 Jul 28;10:12550. doi: 10.1038/s41598-020-69314-2 (PMC7387541; doi:10.1038/s41598-020-69314-2)
Supplement: Supplementary file 1 — Supplementary information. [file 41598_2020_69314_MOESM1_ESM.pdf]

Supplementary Information for

An Unbiased Stereological Method for Corneal Confocal Microscopy in Patients with Diabetic Polyneuropathy

Ellen L. Schaldemose[1], Rasmus E. Hammer[2], Maryam Ferdousi[3,4], Rayaz A. Malik[3,4], Jens R. Nyengaard[2,5], Páll Karlsson[1,2]

[1]Danish Pain Research Center, Aarhus University Hospital, Aarhus, Denmark
[2] Department of Clinical Medicine – Core Center for Molecular Morphology, Section for Stereology and Microscopy**,** Aarhus University, Aarhus, Denmark
[3]Centre for Endocrinology and Diabetes, Institute of Human Development, Manchester Academic Health Science Centre, Manchester, U.K.
[4]Division of Medicine, Weill Cornell Medical College in Qatar, Doha, Qatar
[5]Centre for Stochastic Geometry and Advanced Bioimaging, Aarhus University Hospital, Aarhus, Denmark

Supplementary Table S1. CCM parameters

	**Control (n=26)**	**DSPN(-) (n=45)**	**DSPN(+) (n=17)**	
**Manual**				**Kruskal Wallis**
**CNFD no./mm^2**	34.1 (31.5;36.7)	33.0 (29.8;36.2)	20.8 (14.1;27.5)**^^	**0.002**
**CNFL mm/mm^2**	22.8 (20.3;25.3)	29.9 (23.7;36.0)*	14.8 (10.5;19.1)*^^	**0.006**
**CNBD no./mm^2**	61.3 (55.5;67.1)	52.4 (47:0;57.8)	46.3 (31.8;60.8)	0.096
**Automated**				
**CNFD no./mm^2**	33.9 (31.7;36.1)	28.8 (26.0;31.6)*	18.2 (11.5;25.0)**^	**0.0001**
**CNFL mm/mm^2**	20.6 (19.2;22.0)	17.4 (16.1;18.7)**	13.1 (9.23;16.9)**^	**0.0003**
**CNBD no./mm^2**	44.7 (37.8;51.7)	32.1 (26.5;37.8)*	20.6 (12.4;28.8)**^	**0.0002**
**From original study[a]**				
**IENFD (mean±SD)**	9.8 ± 3.7	7.0 ± 5.0**	5.0 ± 5.5 ** ^^	[a]

Table S1: Mean (95% confidence interval) for the different CCM parameters in the three groups using our new randomized and area adjusted procedure. Results for both the manual and automated analysis method are shown. Possibly differences between the three groups were analyzed by the Kruskal Wallis test due to variations in homogeneity between groups. Significant p-values for Kruskal Wallis test are shown in bold.

DSPN(+) vs DSPN(-): ^p<0.05 ^^p<0.001, unpaired t-test

DSPN(+) vs Control: *p<0.05 **p<0.001, unpaired t-test

DSPN(-) vs Control: *p<0.05 **p<0.001, unpaired t-test

The significant tests presented for the IENFD are calculated in[a]

Supplementary Table S2. Difference in CCM parameters in the adjusted vs unadjusted area.

		Absolute difference (mean (95%CI)) (adjusted – unadjusted)	% mean difference (95%CI)
**Control (n=26)**			
**Manual**			
	**CNFD no./mm²**	8.91 (7.83;9.99)	37 (32;41)%
	**CNFL mm/mm²**	5.84 (5.13; 6.54)	37 (32;41)%
	**CNBD no./mm²**	15.9 (13.9;17.9)	36 (31;41)%
**Automated**			
	**CNFD no./mm²**	8.78 (7.82;9.75)	37 (32;41)%
	**CNFL mm/mm²**	5.38 (4.73;6.04)	37 (32;41)%
	**CNBD no./mm²**	11.4 (9.67;13.2)	34 (30;39)%
**DSPN(-) (n=45)**			
**Manual**			
	**CNFD no./mm²**	10.0 (8.55;11.5)	42 (38;46)%
	**CNFL mm/mm²**	9.81 (6.72;12.9)	43 (39;47)%
	**CNBD no./mm²**	15.7 (13.8;17.6)	40 (36;43)%
**Automated**			
	**CNFD no./mm²**	8.41 (7.58;9.25)	42 (38;46)%
	**CNFL mm/mm²**	5.14 (4.68;5.60)	45 (39;51)%
	**CNBD no./mm²**	9.63 (7.83;11.4)	37 (33;40)%
**DSPN(+) (n=17)**			
**Manual**			
	**CNFD no./mm²**	7.59 (5.04;10.1)	47 (31;64)%
	**CNFL mm/mm²**	5.74 (3.86;7.61)	60 (41;78)%
	**CNBD no./mm²**	19.7 (12.4;26.9)	49 (33;65)%
**Automated**			
	**CNFD no./mm²**	6.83 (4.27;9.39)	49 (32;64)%
	**CNFL mm/mm²**	5.16 (3.29;7.02)	64 (46;83)%
	**CNBD no./mm²**	8.18 (4.18;12.2)	37 (24;50)%

Table S2: Larger values after adjusted for area in focus

Supplementary Figure S1. CCM parameters, new sampling method with adjusted area.

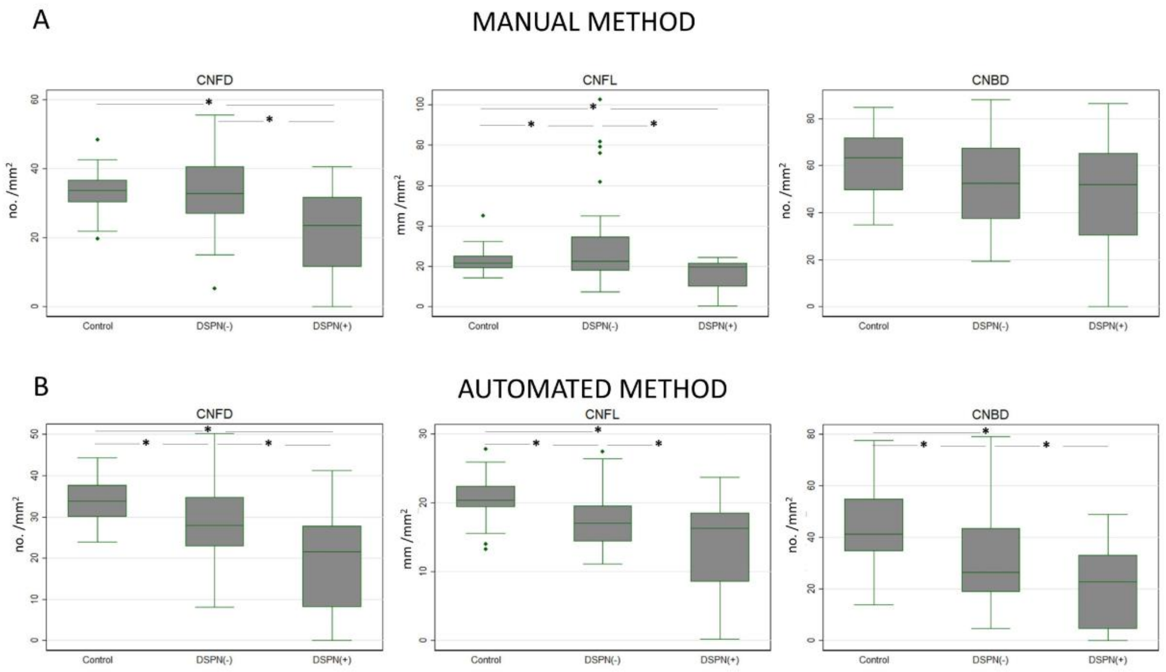

Figure S1. a and b. Boxplot of CNFD, CNFL and CNBD in the three groups using the new randomized and area adjusted method. Horizontal line: median CCM value,
boxes: interquartile range, whiskers: adjacent values.
Dots are outside values. * p<0.05
a: manual CCM analysis. b: automated CCM analysis.

Supplementary Figure S2. Comparison between CCM results using the randomized sampling method and adjusted and standard areas for manual and automated analysis.

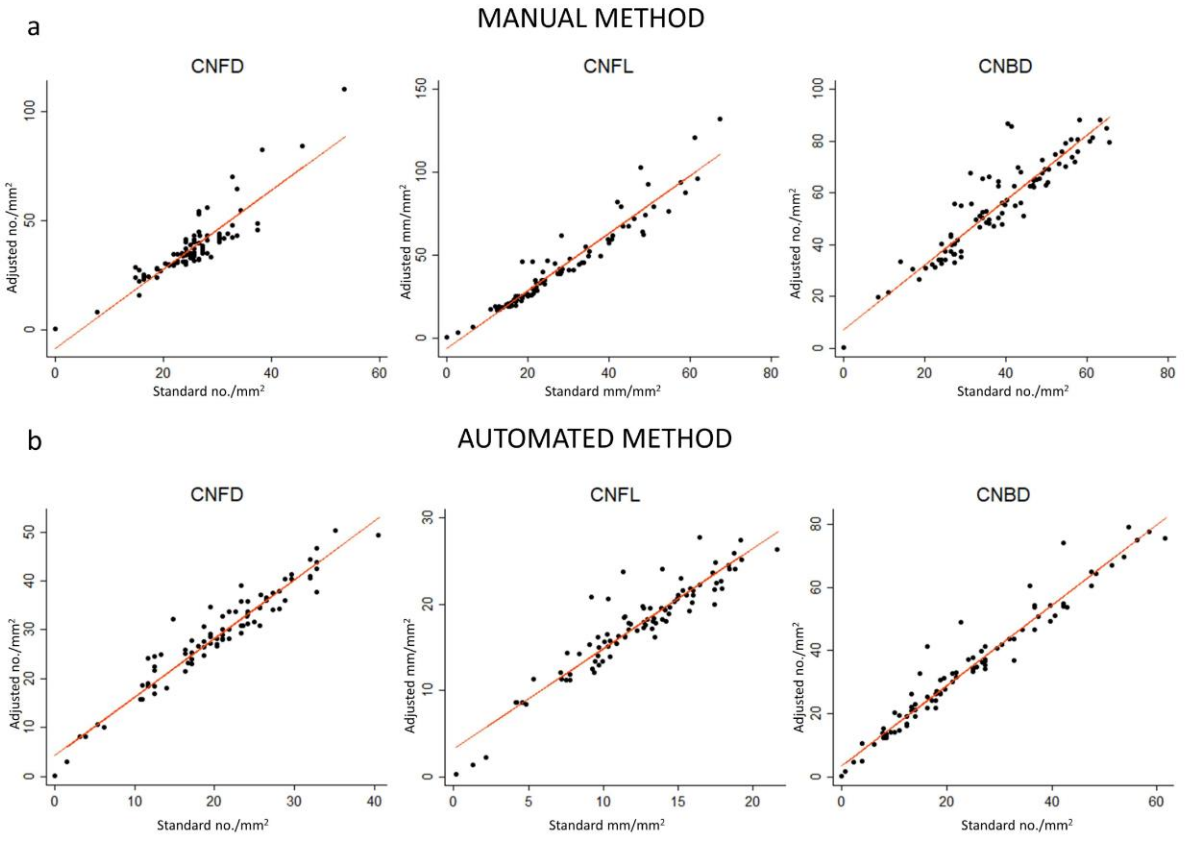

Supplementary Figure S2. a and b. Comparison between areas, CNFD, CNFL and CNBD. Scatter plots and identity lines between the adjusted and standard area, grouped by method of analysis (a: manual, b: automated). The dots represent the mean values from the individual participants and the dashed lines indicate the identity line. The Pearson's correlation coefficient were r = 0.89 (manual method) and r = 0.97 (automated method) for the CNFD values, r = 0.96 (manual method) and r = 0.93 (automated method) for the CNFL values and r = 0.93 (manual method) and r = 0.97 (automated method) for the CNBD values.

References - supplementary

[a] Chen, X. *et al.* Small nerve fiber quantification in the diagnosis of diabetic sensorimotor polyneuropathy: comparing corneal confocal microscopy with intraepidermal nerve fiber density. *Diabetes care* **38**, 1138-1144, doi:10.2337/dc14-2422 [doi] (2015).
